# Drought Tolerance Is Correlated with the Activity of Antioxidant Enzymes in* Cerasus humilis* Seedlings

**DOI:** 10.1155/2016/9851095

**Published:** 2016-03-07

**Authors:** Jing Ren, Li Na Sun, Qiu Yan Zhang, Xing Shun Song

**Affiliations:** ^1^Department of Genetics, College of Life Science, Northeast Forestry University, Harbin 150040, China; ^2^Key Laboratory of Dairy Science, Ministry of Education, College of Food Science, Northeast Agricultural University, Synergetic Innovation Center of Food Safety and Nutrition, Harbin, Heilongjiang 150030, China; ^3^State Key Laboratory of Tree Genetics and Breeding, Northeast Forestry University, Harbin 150040, China

## Abstract

*Cerasus humilis*, grown in the northern areas of China, may experience water deficit during their life cycle, which induces oxidative stress. Our present study was conducted to evaluate the role of oxidative stress management in the leaves of two* C. humilis* genotypes, HR (drought resistant) and ND4 (drought susceptible), when subjected to a long-term soil drought (WS). The HR plants maintained lower membrane injury due to low ROS and MDA accumulation compared to ND4 plants during a long-term WS. This is likely attributed to global increase in the activities of superoxide dismutase (SOD) isoenzymes and enzymes of the ascorbate-glutathione (AsA-GSH) cycle and maintenance of ascorbate (AsA) levels. Consistent closely with enzymes activities, the expression of cytosolic ascorbate peroxidase (cAPX) and dehydroascorbate reductase (DHAR) followed a significant upregulation, indicating that they were regulated at the transcriptional level for HR plants exposed to WS. In contrast, ND4 plants exhibited high ROS levels and poor antioxidant enzyme response, leading to enhanced membrane damage during WS conditions. The present study shows that genotypic differences in drought tolerance could be likely attributed to the ability of* C. humilis* plants to induce antioxidant defense under drought conditions.

## 1. Introduction

Drought is one of the most important manifestations of abiotic stress in plants. It is the major yield-limiting factor of crop plants and it actively and continuously determines the natural distribution of plant species [1]. To meet the needs of the growing world population, it is essential to effectively utilize dehydrated soil in drought-prone areas. However, the progress towards developing drought-tolerant crops is significantly hampered by the lack of highly tolerant genetic resources and the complexity in physiological and genetic traits. It is therefore important to identify the genetic resources and to understand the mechanisms of drought tolerance in plants that could result in high levels of tolerance to drought stress [2]. Plants evolve adaptations to different growth conditions and there exist great differences in their tolerance towards a variety of growth conditions. In fact, great differences are also observed within species, since different cultivars suited for different ecosystems or growing seasons have been developed by breeders [3].

Reactive oxygen species (ROS), also called active oxygen species (AOS) or reactive oxygen intermediates (ROI), are the result of the partial reduction of atmospheric O_2_. Although the role of ROS production and control during drought stress is yet to be resolved, ROS seem to have a dual effect under abiotic stress conditions that depend on their overall cellular amount. If kept at relatively low levels they are likely to function as components of a stress-signaling pathway, triggering stress defense/acclimation responses. However, when reaching a certain level of phytotoxicity ROS become extremely deleterious, initiating uncontrolled oxidative cascades that damage cellular membranes and other cellular components resulting in oxidative stress and eventually cell death [4–6]. Plants have also developed strategies to minimize the deleterious effects of ROS which cause lipid peroxidation, protein denaturation, and DNA mutation [7]. Among them, the ROS-scavenging mechanism has received an increasing amount of attention. This mechanism consists of such enzymes as superoxide dismutase (SOD) and catalase (CAT) and the enzymes of the ascorbate-glutathione cycle (e.g., ascorbate peroxidase (APX), monodehydroascorbate reductase (MDAR), dehydroascorbate reductase (DHAR), and glutathione reductase (GR)) and nonenzymatic components such as ascorbate, glutathione, carotenoids, and tocopherol [8–10]. It is known that organelles with a highly oxidizing metabolic activity or an intense rate of electron flow, such as chloroplasts, mitochondria, and microbodies, are major sources of ROS. In accordance with this, different isoenzymes such as Cu/Zn-SOD, Fe-SOD, Mn-SOD, cytosol APX (cAPX), and microbody APX (mAPX) have been found in different organelles [11]. Although it has been generalized that the tolerance of a species to different stresses is closely correlated with its ROS-scavenging capacity [12], other tolerance (or avoidance) mechanisms have been suggested to modify genotype-dependent responses to stress, along with the different degrees of stress experienced, species, and plant ages [13–15].

*Cerasus humilis* (Bge.) Sok is a species of small, perennial, deciduous shrub belonging to Rosaceae family. It is distributed in the Inner Mongolia, Shanxi, and the northern areas of China.* C. humilis* fruits contain a variety of mineral elements beneficial to human health, especially a higher calcium content of fruit. In the early seedling periods, however,* C. humilis* not only grows slowly but is vulnerable to the environmental changes [16, 17]. Our previous studies found contrasting* C. humilis* genotypes Huai'rou (HR, drought-tolerant) and Nongda4 (ND4, drought-sensitive) in response to drought stress. However, the basic physiological, biochemical, and molecular mechanisms involved in stress tolerance are still unclear. The main objective of the present study was to compare the physiological effects of drought stress on the two* C. humilis* genotypes and to test the hypothesis that genotypic differences in growth response to drought are related to ROS-scavenging activity.

## 2. Material and Methods

### 2.1. Plant Material

Two* C. humilis* genotypes were used in this study. Huai'rou (HR) is a drought-tolerant genotype developed for arid areas while Nongda4 (ND4) is a drought-sensitive genotype developed for humid areas. All cuttings of 3-year-old plants were cut at the beginning of March in 2012, then transplanted into a container (35 × 35 × 25 cm) filled with organic soil, and irrigated regularly by half-strength Enshi nutrient solution under a 12 h photoperiod at temperatures ranging from about 17 to 25°C, photosynthetic photon flux density (PPFD) of 60 *μ*mol (photon) m^−2^ s^−1^, and the relative humidity of 70–75% in the greenhouse. At the end of May in 2015, plants at the 35–40-leaf stage were randomly allocated to one of two treatments: control plants (control) were watered daily to field capacity, while water was withheld from water-stressed plants (drought). The drought treatment lasted for 21 d; two mature leaves for each plant were removed and quickly frozen in liquid nitrogen, stored at −80°C for subsequent measurements of all physiological and biochemical parameters, with at least 30 plants per treatment.

Relative growth rate (RGR) was calculated by

(1) $$\begin{array}{c} RGR=\frac{\left(lnW_{2}-lnW_{1}\right)}{t_{2}-t_{1}}, \end{array}$$

where *W*_2_ and *W*_1_ were dry weight for seedlings at the beginning and at the end of the experiment, respectively, while *t*_2_ − *t*_1_ was the time duration for the treatment [18].

### 2.2. Determination of Reactive Oxygen Species and Lipid Peroxidation Level

O_2_^∙−^ was measured by monitoring nitrite formation from hydroxylamine in the presence of O_2_^∙−^, as described in [19] with slight modifications. Each 0.5 g of frozen leaf segment was homogenized in 3 mL of 65 mm potassium phosphate buffer (pH 7.8) and centrifuged at 5000 g for 10 min. The incubation mixture contained 0.9 mL of 65 mm phosphate buffer (pH 7.8), 0.1 mL 10 mm hydroxylamine hydrochloride, and 1 mL of the supernatant. Absorbance in the aqueous solution was measured at 530 nm. H_2_O_2_ content was measured by monitoring A_410_ of a titanium-peroxide complex, following the method described by [20]. As for the lipid peroxidation level, measured as the content of malondialdehyde (MDA), leaves were homogenized with 5 mL of 50 mM solution containing 0.07% NaH_2_PO_4_·2H_2_O and 1.6% Na_2_HPO_4_·12H_2_O and centrifuged at 20,000 ×g for 25 min in a refrigerated centrifuge. For measurement of MDA concentration, method of Heath and Packer was used [21].

### 2.3. Assay of SOD Isoenzymes Activities

To determine the activities of antioxidant enzymes, a crude enzyme extract was prepared by homogenizing 500 mg of leaf tissue in extraction buffer (0.5% Triton X-100 and 1% polyvinylpyrrolidone in 100 mM potassium phosphate buffer, pH 7.0) using a chilled mortar and pestle. The homogenate was then centrifuged at 15,000 ×g for 20 min at 4°C, and the supernatant was used for the enzymatic assays described below. SOD (EC 1.15.1.1) activity was assayed by monitoring the inhibition of photochemical reduction of nitro blue tetrazolium (NBT), according to [22]. Activities of different forms of SOD were identified by adding KCN and/or H_2_O_2_ in the reaction mixture [23]. KCN inhibits Cu/Zn-SOD but does not affect Mn-SOD nor Fe-SOD, whereas H_2_O_2_ inactivates Cu/Zn-SOD and Fe-SOD without affecting Mn-SOD. In addition, peroxidases might interfere with the SOD assay in the presence of exogenous H_2_O_2_ [24]. After extensive preliminary testing of a range of concentrations, KCN was added to the reaction mixture to a final concentration of 3 mM before the addition of H_2_O_2_ to a final concentration of 5 mM to eliminate interference from peroxidase and catalase enzymes [25].

### 2.4. Assay for APX-GSH Cycle Enzymes Activities and Contents of AsA and GSH

APX (EC1.11.1.11) activity was determined by monitoring the decrease in A_290_ according to [26]. MDAR (EC1.6.5.4) activity was measured by monitoring the decrease in A_340_ due to the NADH oxidation [27]. DHAR (EC1.8.5.1) was measured according to [28]. GR (EC1.6.4.2) was determined following the procedure described by [29]. CAT (EC 1.11.1.6) activity was assayed in a reaction mixture containing 25 mM phosphate buffer (pH 7.0), 10 mM H_2_O_2_, and the enzyme. The decomposition of H_2_O_2_ was followed at 240 nm [30]. Ascorbate (AsA) and GSH content were determined according to [31, 32], respectively.

### 2.5. qRT-PCR Analysis

For qRT-PCR, duplicate samples were analysed in a Quantitative PCR instrument (Roche LightCycler 480 II, Switzerland). Total RNA was prepared using Trizol; then RNA was treated with RNase-free DNase I according to the manufacturer's instruction. One-microgram total RNA was performed in reverse transcription with RevertAid Reverse Transcriptase (Fermentas) and Oligo d(T)primers (TaKaRa). PCR amplification was performed with 40 cycles as follows: 94°C for 30 s, 58°C for 30 s, and 72°C for 30 s, followed by 72°C for 10 min. The relative expression levels of genes were presented by 2^−ΔΔCT^. PCR reactions employed the following primers, actin-F (5′-GTGAAGGCTGGGTTTGCT-3′), actin-R (5′-CCCATCCCAACCATAACA-3′), DHAR-F (5′-CTCCTCCACCATCAAACA-3′), DHAR-R (5′-TTAGCCAAGTCCACCAAC-3′), cAPX-F (5′-AGCCCATCAAGCAACAGT-3′), and cAPX-R (5′-AGGGTCCTTCAAATCCAG-3′), respectively.

### 2.6. Statistic Analysis

Data are the average of at least three independent replicates. ANOVA, using* PC SAS* version* 8.2* (*SAS Institute*, Cary, NC, USA), was conducted for all data and Duncan's Multiple-Range Test (DMRT) was used to evaluate treatment effects (*P* < 0.05) by using the data processing system statistical software package.

## 3. Results

### 3.1. Growth Response

**Figure 1:**
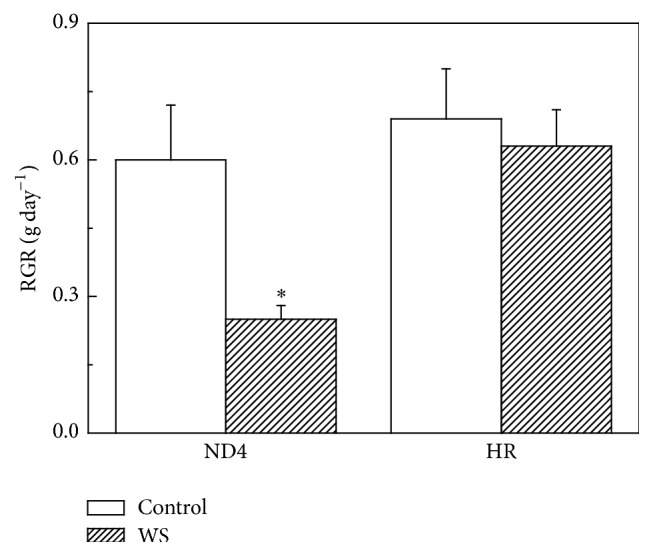
Effects of water stress (WS) on relative growth rate (RGR) as measured by dry matter production of* Cerasus humilis* leaves of Huai'rou (HR) and Nongda4 (ND4). Samples were collected after 21 d of treatment. The data are the mean of at least three replicates with standard errors shown by vertical bars. Asterisk (*∗*) indicates significant difference with control groups (well-watered) at the 0.05 level of probability by Duncan's Multiple-Range Test.

First of all, we investigated changes in the relative growth rate (RGR) of two genotypes in response to WS (Figure 1). There was no significant difference between the two genotypes in RGR for* C. humilis* plants under control conditions. However, exposure to WS resulted in a significant reduction in RGR in ND4 from 0.9 to 0.6 g day^−1^. In sharp contrast, no such decrease in RGR was observed for HR plants (Figure 1).

### 3.2. ROS Generation and Lipid Peroxidation

**Figure 2:**
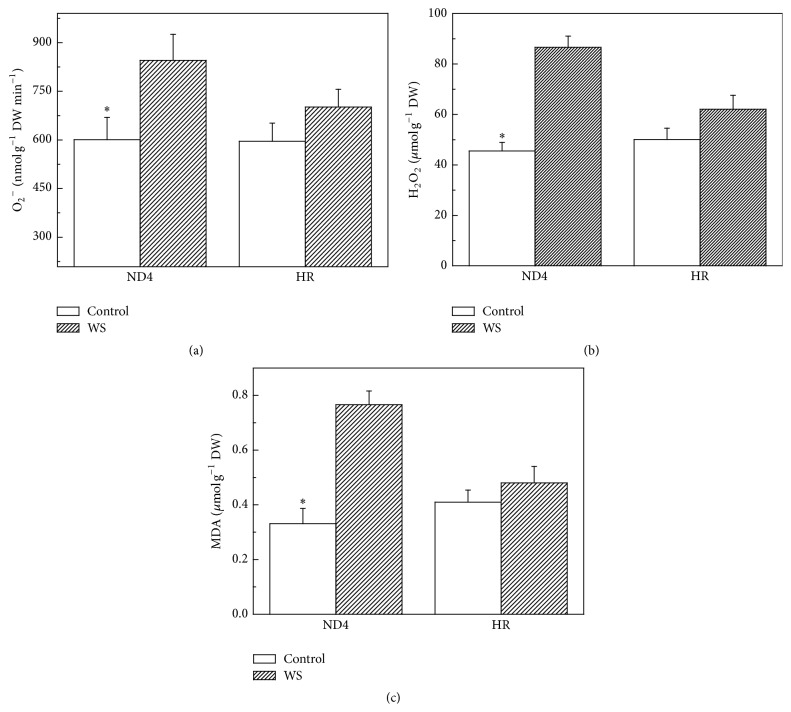
Effects of water stress (WS) on O_2_^∙−^ (a), H_2_O_2_ (b), and lipid peroxidation (expressed as malondialdehyde, MDA) (c) in* Cerasus humilis* leaves of Huai'rou (HR) and Nongda4 (ND4). Samples were collected after 21 d of treatment. The data shown are the mean of at least three replicates with standard errors shown by vertical bars. Asterisk (*∗*) indicates significant difference with control groups (well-watered) at the 0.05 level of probability by Duncan's Multiple-Range Test.

Exposure to WS resulted in O_2_^∙−^ production rate changes between the two* C. humilis* genotypes. The rate increased by 36% for ND4 while no significant change was observed for HR (Figure 2(a)). Meanwhile, HR plants showed a lower H_2_O_2_ content compared to ND4 when they were grown under WS. WS resulted in a 13% increase in H_2_O_2_ content in ND4 but not in HR (Figure 2(b)).

The lipid peroxidation level in the leaves grown under either WS or control conditions, measured as the content of malondialdehyde (MDA), is given (Figure 2(c)). Similar to the changes in H_2_O_2_ content, MDA content significantly increased in ND4 plants after exposure to a long-term WS while it was independent of the drought condition in HR seedlings.

### 3.3. SOD Activity

**Figure 3:**
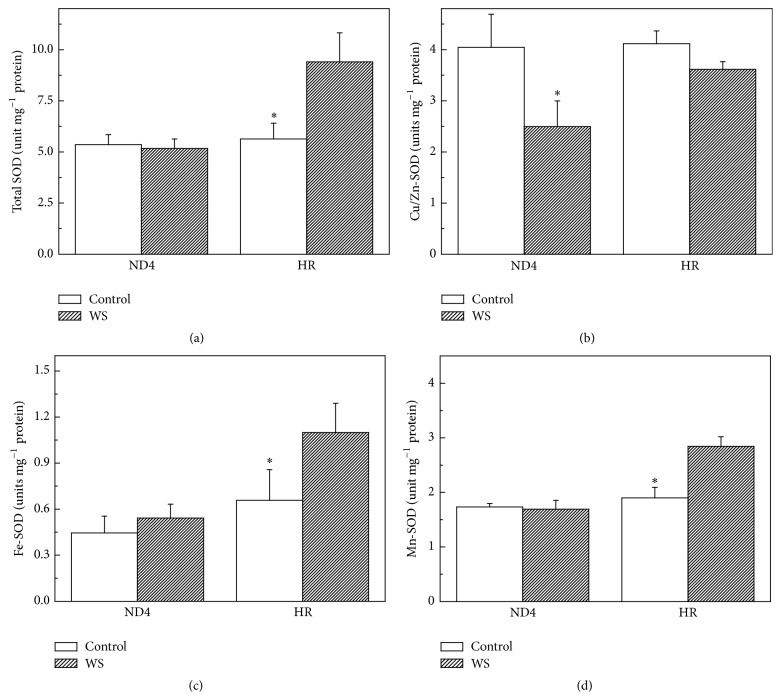
Effects of water stress (WS) on the activities of total SOD (a) and SOD isoenzymes including Cu/Zn-SOD (b), Fe-SOD (c), and Mn-SOD (d) in* Cerasus humilis* leaves of Huai'rou (HR) and Nongda4 (ND4). Samples were collected after 21 d of treatment. The data shown are the mean of at least three replicates with standard errors shown by vertical bars. Asterisk (*∗*) indicates significant difference with control groups (well-watered) at the 0.05 level of probability by Duncan's Multiple-Range Test.

Total SOD activity remained almost unchanged for both genotypes throughout the experiment when the plants were grown at optimal growth conditions. However, a 67% increase in SOD activity was observed in HR plants under WS. In contrast, no significant increase was found in ND4 plants exposed to WS (Figure 3(a)).

A detailed examination was carried out to determine the response of three SOD isoenzymes to WS. There were no significant differences in the activity of Cu/Zn-SOD between the two genotypes (Figure 3(b)), while Fe-SOD and Mn-SOD were a little higher (but not significant) in HR than in ND4 for plants grown in optimal growth conditions (Figures 3(c) and 3(d)). A long-term WS resulted in a significant increase in Mn-SOD and Fe-SOD activity but not in Cu/Zn-SOD activity for HR plants. In contrast, there was a remarkable decrease in Cu/Zn-SOD activity for ND4 plants. WS had little effect on the activities of the Mn-SOD and Fe-SOD in ND4 plants (Figures 3(b), 3(c), and 3(d)).

### 3.4. CAT and AsA-GSH Cycle System Enzymes

**Figure 4:**
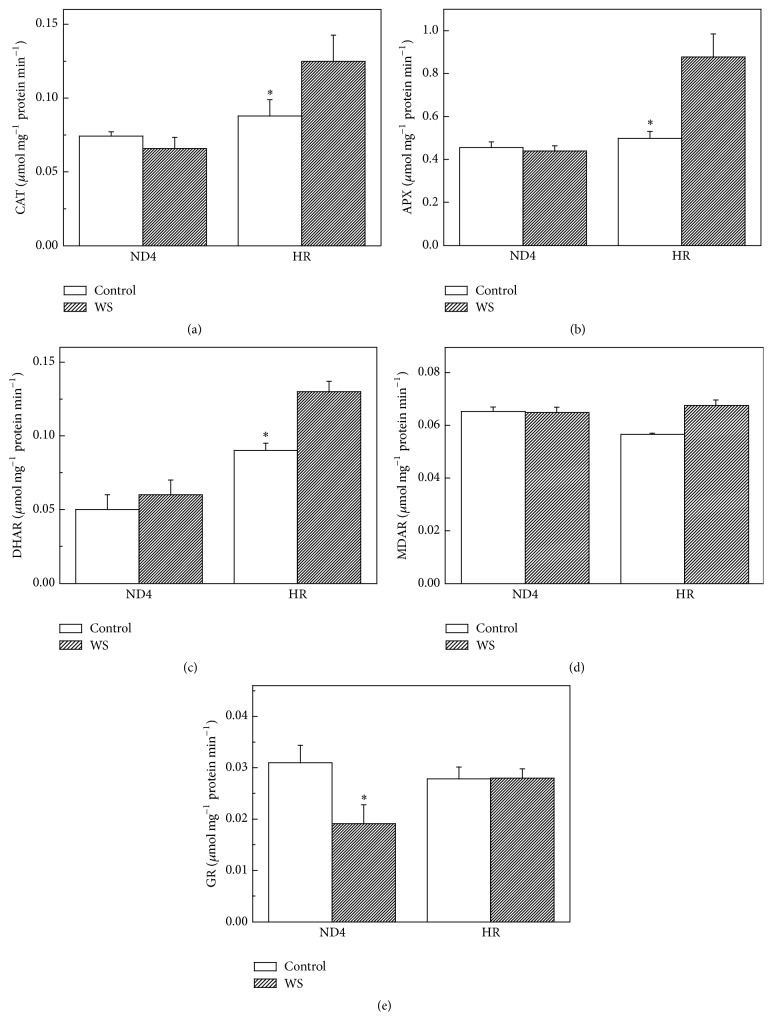
Effects of water stress (WS) on the activities of catalase (CAT) (a) and ascorbate-glutathione (AsA-GSH) cycle enzymes including ascorbate peroxidase (APX) (b), dehydroascorbate reductase (DHAR) (c), monodehydroascorbate reductase (MDAR) (d), and glutathione reductase (GR) (e) in* Cerasus humilis* leaves of Huai'rou (HR) and Nongda4 (ND4). Samples were collected after 21 d of treatment. The data are the mean of at least three replicates with standard errors shown by vertical bars. Asterisk (*∗*) indicates significant difference with control groups (well-watered) at the 0.05 level of probability by Duncan's Multiple-Range Test.

Similar to the changes in total SOD activity, exposure to a long-term WS led to a significant increase in CAT activity in HR plants. However, for ND4 plants, there were no changes in CAT activity under either WS or control conditions (Figure 4(a)). APX activity showed changes very similar to CAT, increasing under WS in HR but not in ND4 plants (Figure 4(b)). For HR plants, WS resulted in a sharp increase by 79% in DHAR activity under WS. There was no significant difference in MDAR and GR activities under either WS or control conditions. For ND4 plants, however, a decrease by 63% in GR activities, with no changes in MDAR and DHAR activities, was observed under WS compared to the control plants (Figures 4(c), 4(d), and 4(e)).

### 3.5. AsA and GSH Levels

**Figure 5:**
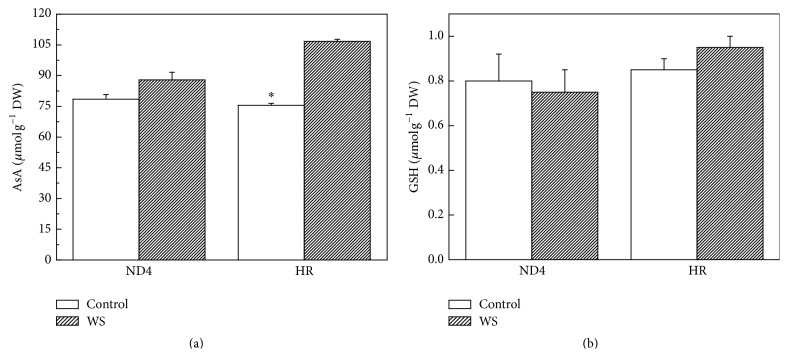
Effect of water stress (WS) on ascorbate (AsA) (a) and glutathione (GSH) (b) contents in* Cerasus humilis* leaves of Huai'rou (HR) and Nongda4 (ND4). Samples were collected after 21 d of treatment. The data are the mean of at least three replicates with standard errors shown by vertical bars. Asterisk (*∗*) indicates significant difference with control groups (well-watered) at the 0.05 level of probability by Duncan's Multiple-Range Test.

As regards antioxidant contents, there were no significant differences in AsA contents between the two genotypes when grown under control conditions. However, WS induced higher AsA contents for HR (41%) than that for ND4 plants (12%). In comparison, WS had no measurable effect on GSH contents for both genotypes under either WS or control conditions (Figure 5).

### 3.6. cAPX and DHAR Transcript Levels

**Figure 6:**
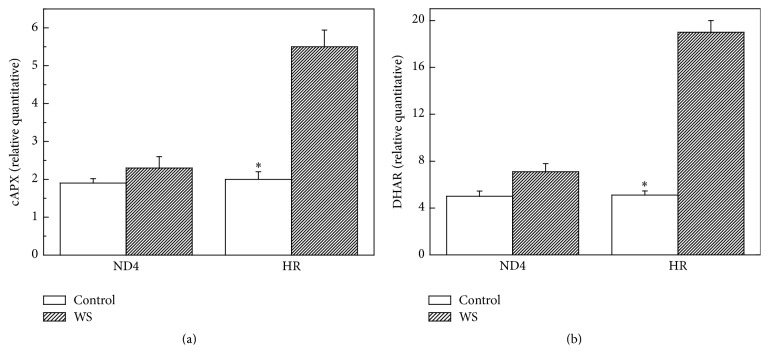
Effects of water stress (WS) on expression pattern of cytosol APX (cAPX) (a) and dehydroascorbate reductase (DHAR) (b) in* Cerasus humilis* leaves of Huai'rou (HR) and Nongda4 (ND4) by qRT-PCR. Data are the means of at least five replicates with standard errors shown by vertical bars. Asterisk (*∗*) indicates significant difference with control groups (well-watered) at the 0.05 level of probability by Duncan's Multiple-Range Test.

Furthermore, to examine the change in expression levels for some antioxidative enzymes in both* C. humilis* genotypes during WS, transcript levels of DHAR and cAPX genes were analysed by qRT-PCR in the same samples as used in the enzyme activity analysis. A sharp increase in transcript level of DHAR and cAPX was observed for HR plants under WS-period. However, for ND4, there were no changes in DHAR and cAPX expressions between treated and control seedlings (Figures 6(a) and 6(b)).

## 4. Discussion

Water stress is considered a detrimental factor for the production of crops worldwide. Thus, it is imperative to identify genetic resources tolerant to drought stress in an effort to stabilize agricultural production [2]. The present study was carried out to elucidate the physiological mechanism of two* C. humilis* genotypes by comparatively examining growth parameters, the O_2_^∙−^ and H_2_O_2_ concentrations, the level of lipid peroxidation, ROS metabolism, and gene expression responses.

In the present study, HR plants exhibited a higher relative growth rate than ND4 plants after exposure to WS (Figure 1), indicating that HR is more drought-tolerant than ND4. HR appears to have acquired greater adaptation to water shortage compared to ND4. ND4 plants show a more significant accumulation of ROS (Figures 2(a) and 2(b)), indicating that the oxidative stress occurred. A relatively less increase of ROS in HR implied that antioxidative system was likely, at least in part, involved in balancing the ROS levels. As an indicator of membrane lipid peroxidation, the MDA content of tolerant genotype HR showed less elevation under WS than in ND4 plants (Figure 2(c)), suggesting that the recovery ability of the tolerant genotype was higher than the sensitive genotype. Under stress conditions, the level of MDA accumulation is different in plant genotypes with contrasting tolerance. The formed MDA is capable of reacting with free amino groups of proteins and phospholipid components and initiating the appearance of ethylene in membranes. This may lead to alterations of the properties of whole membrane and also of the individual cell components under stress [33, 34].

SOD is one of the key components of the cell protection system against oxidative stress. It is known that SOD has three different isoenzymes distributed between different organelles. Cu/Zn-SOD is mostly located in chloroplasts, cytosol, and peroxisomes, while Fe-SOD and Mn-SOD are found mostly in chloroplasts and mitochondria, respectively [11]. As several isoforms of SOD differently contributing to the total activity of the enzyme are present in* C. humilis* leaves, it was important to assess the contribution of each isoform to the total activity of the enzyme under drought. Our results on SOD isoenzyme activities (Figure 3) suggested that Fe-SOD and Mn-SOD could play the main role in detoxification of superoxide radicals in chloroplasts and mitochondria. Similar report has been shown in wheat varieties subjected to continuous soil drought [34]. Indirect evidence has been reported by Zhang et al. who found that overexpression of* Tamarix albiflonum TaMnSOD* increases drought tolerance in transgenic cotton [35]. The decrease of Cu/Zn-SOD activity for ND4 plants exposed to WS (Figure 3(b)) was likely attributed to the influence of H_2_O_2_. Similar results have been reported by Smirnoff [36].

CAT and APX in the AsA-GSH cycle enzymes are responsible for the decomposition of H_2_O_2_ generated by SOD in different cellular organelles. We found that the activity of CAT and APX showed similar patterns of change to that observed for SOD activity in HR plants (Figures 4(a) and 4(b)), suggesting that CAT and APX work in a coordinated manner to scavenge H_2_O_2_. The maintenance of CAT activity in leaves of drought-stressed plants likely allowed the removal of photorespiratory H_2_O_2_ produced when plants are subjected to water deficit, especially under severe degrees of stress [37]. An analysis of the recent literature pointed out that an increase in CAT activity is generally positively related to the degree of drought experienced by plants [38–40] and that APX plays a positive key role in drought stress responses and following recovery from drought [38, 41, 42].

DHAR activity was increased significantly for HR plants exposed to WS (Figure 4(c)), indicating that DHAR is responsible for AsA regeneration in plant tissues. The results, along with the increased expressions of DHAR and cAPX (Figure 6), suggested that the two antioxidant enzymes were regulated in the transcript levels. In sharp contrast with the changes in SOD, APX, and DHAR activity, GR activity decreased in ND4 plants after the exposure to WS (Figure 4(e)). This decrease could be attributed to reduced NADPH availability since stress usually results in a decrease in the supply of reductants such as ATP and NADPH. It is also possible that ROS accumulation resulted in the inhibition of enzymes involved in the ROS-scavenging system [43]. Furthermore, our results also found that WS induced greater increases in AsA level in HR plants (Figure 5(a)), implying that it was likely involved in the operation of the ROS detoxification machinery assumed by APX/GSH cycle [44, 45].

In a brief conclusion, our findings demonstrate that the oxidative stress is differentially expressed in ND4 and HR in response to drought stress, with higher tolerance exhibited by the latter.
